# Supplementary material for: Resolving the full spectrum of human genome variation using Linked-Reads
Source: Genome Res. 2019 Apr;29(4):635–45. doi: 10.1101/gr.234443.118 (PMC6442396; doi:10.1101/gr.234443.118)
Supplement: Supplemental Material [file supp_gr.234443.118_Supplemental_longranger-2.2.zip › longranger-2.2.tar.bz2!/lr2.2-public/lib/stan/cmdstan/stan/doc/api/html/init__static__hmc_8hpp_source.html]

Stan: src/stan/common/init\_static\_hmc.hpp Source File


|  |  |
| --- | --- |
|  | Stan  2.5.0  probability, sampling & optimization |


- Main Page
- Related Pages
- Namespaces
- Classes
- Files

- File List
- File Members

All Classes Namespaces Files Functions Variables Typedefs Enumerations Enumerator Friends Macros Pages

- src
- stan
- common

init\_static\_hmc.hpp

Go to the documentation of this file.

1 #ifndef STAN\_\_COMMON\_\_INIT\_STATIC\_HMC\_HPP

2 #define STAN\_\_COMMON\_\_INIT\_STATIC\_HMC\_HPP

3

4 #include <stan/mcmc/base\_mcmc.hpp>

5 #include <stan/gm/arguments/argument.hpp>

6 #include <stan/gm/arguments/categorical\_argument.hpp>

7 #include <stan/gm/arguments/singleton\_argument.hpp>

8

9 namespace stan {

10  namespace common {

11

12  template<class Sampler>

13  bool init\_static\_hmc(stan::mcmc::base\_mcmc\* sampler,

14  stan::gm::argument\* algorithm) {

15

16  stan::gm::categorical\_argument\* hmc

17  = dynamic\_cast<stan::gm::categorical\_argument\*>

18  (algorithm->arg("hmc"));

19

20  stan::gm::categorical\_argument\* base

21  = dynamic\_cast<stan::gm::categorical\_argument\*>

22  (algorithm->arg("hmc")->arg("engine")->arg("static"));

23

24  double epsilon

25  = dynamic\_cast<stan::gm::real\_argument\*>

26  (hmc->arg("stepsize"))->value();

27  double epsilon\_jitter

28  = dynamic\_cast<stan::gm::real\_argument\*>

29  (hmc->arg("stepsize\_jitter"))->value();

30  double int\_time

31  = dynamic\_cast<stan::gm::real\_argument\*>(base->arg("int\_time"))->value();

32

33  dynamic\_cast<Sampler\*>(sampler)->set\_nominal\_stepsize\_and\_T(epsilon, int\_time);

34  dynamic\_cast<Sampler\*>(sampler)->set\_stepsize\_jitter(epsilon\_jitter);

35

36  return true;

37  }

38

39  }

40 }

41

42 #endif

base\_mcmc.hpp

categorical\_argument.hpp

stan::gm::singleton\_argument

**Definition:** singleton\_argument.hpp:44

stan::gm::categorical\_argument::arg

argument \* arg(const std::string name)

**Definition:** categorical\_argument.hpp:143

argument.hpp

stan::gm::categorical\_argument

**Definition:** categorical\_argument.hpp:11

stan::gm::argument::arg

virtual argument \* arg(const std::string name)

**Definition:** argument.hpp:67

singleton\_argument.hpp

stan::gm::argument

**Definition:** argument.hpp:12

stan::common::init\_static\_hmc

bool init\_static\_hmc(stan::mcmc::base\_mcmc \*sampler, stan::gm::argument \*algorithm)

**Definition:** init\_static\_hmc.hpp:13

stan::mcmc::base\_mcmc

**Definition:** base\_mcmc.hpp:13


---

[ Stan Home Page ]

*© 2011–2014,
Stan Development Team.*
